# Sensory Dysfunction of Bladder Mucosa and Bladder Oversensitivity in a Rat Model of Metabolic Syndrome

**DOI:** 10.1371/journal.pone.0045578

**Published:** 2012-09-19

**Authors:** Wei-Chia Lee, Po-Hui Chiang, You-Lin Tain, Chia-Ching Wu, Yao-Chi Chuang

**Affiliations:** 1 Division of Urology, Kaohsiung Chang Gung Memorial Hospital and Chang Gung University College of Medicine, Kaohsiung, Taiwan; 2 Department of pediatrics, Kaohsiung Chang Gung Memorial Hospital and Chang Gung University College of Medicine, Kaohsiung, Taiwan; 3 Department of International Business, College of Commerce and Management, Cheng Shiu University, Kaohsiung, Taiwan; Max-Delbrück Center for Molecular Medicine (MDC), Germany

## Abstract

**Purpose:**

To study the role of sensory dysfunction of bladder mucosa in bladder oversensitivity of rats with metabolic syndrome.

**Materials and Methods:**

Female Wistar rats were fed a fructose-rich diet (60%) or a normal diet for 3 months. Based on cystometry, the fructose-fed rats (FFRs) were divided into a group with normal detrusor function or detrusor overactivity (DO). Acidic adenosine triphosphate (ATP) solution (5mM, pH 3.3) was used to elicit reflex micturition. Cystometric parameters were evaluated before and after drug administration. Functional proteins of the bladder mucosa were assessed by western blotting.

**Results:**

Compared to the controls, intravesical acidic ATP solution instillation induced a significant increase in provoked phasic contractions in both FFR groups and a significant decrease in the mean functional bladder capacity of group DO. Pretreatment with capsaicin for C-fiber desentization, intravesical liposome for mucosal protection, or intravenous pyridoxal 5-phosphate 6-azophenyl-2′,4′-disulfonic acid for antagonized purinergic receptors can interfere with the urodynamic effects of intravesical ATP in FFRs and controls. Over-expression of TRPV1, P2X_3_, and iNOS proteins, and down-regulation of eNOS proteins were observed in the bladder mucosa of both fructose-fed groups.

**Conclusions:**

Alterations of sensory receptors and enzymes in the bladder mucosa, including over-expression of TRPV1, P2X_3_, and iNOS proteins, can precipitate the emergence of bladder phasic contractions and oversensitivity through the activation of C-afferents during acidic ATP solution stimulation in FFRs. The down-regulation of eNOS protein in the bladder mucosa of FFRs may lead to a failure to suppress bladder oversensitivity and phasic contractions. Sensory dysfunction of bladder mucosa and DO causing by metabolic syndrome are easier to elicit bladder oversensitivity to certain urothelium stimuli.

## Introduction

BO (bladder oversensitivity), also referred to “increased bladder sensation” previously, can be defined as increased perceived bladder sensation during bladder filling with a low maximum cystometric bladder capacity by the International Continence Society in 2010 [1]. BO resulting from afferent noise is one of the important factors causing an overactive bladder (OAB) [2]. Recently, scientists have indicated that people with metabolic syndrome have a higher prevalence of lower urinary tract symptoms, particularly OAB symptoms [3].

Evidence suggests increased expression or release of sensory receptors or neurotransmitters in the mucosal layer of the bladder, including the urothelial layer and suburothelial layer, can generate afferent noise via C fiber pathway and result in OAB [2], [4]. Desensitizing suburothelial afferent nerves or blocking the urothelial functional receptors (such as TRPV1 receptor antagonist) is a potential treatment for OAB [5]. The urothelium expresses transient receptor potential vanilloid 1 (TRPV1) and P2X_3_ receptors. It also releases adenosine triphosphate (ATP) and nitric oxide (NO) and can activate C-fiber afferents [2], [4]. Over-expression of TRPV1 [6] and P2X_3_ receptors [7] of urothelium and hypersensitivity of C-fiber pathway [8] are associated with urgency and detrusor overactivity (DO) in humans. In addition, DO is associated with increased ATP and decreased constitutive NO release from the urothelium of streptozocin-induced diabetic rats [9].

Intravesical administration of acidic ATP solution can provide protons and ATP for acting on TRPV1 receptors, purinergic receptors, and enhance ATP penetration through urothelium in an acidic environment [10], [11]. This may activate C-fiber afferents, increase sensory activity, and enhance bladder overactivity [12]. The TRPV1 receptor is an integrator of thermal, noxious chemical and acidic stimuli. It also regulates the frequency of bladder reflex contractions, either through direct excitation of sensory fibers or through urothelial-sensory fiber cross talk involving the release of neuromediators from the urothelial cells [2], [4]. The P2X_3_ receptor can be expressed as P2X_2_/P2X_3_ heteromer or P2X_3_ homomer in the bladder [13], [14]. Extracellular ATP release from activation of TRPV1 or from stretching may increase the excitability of bladder afferents through the P2X_3_ receptors to facilitate bladder reflex activity [2], [4].

**Table 1:** Comparisons of general characteristics, biochemistry variables, and cystometric parameters among controls and FFRs.

	Controls	FFRs with NDF	FFRs with DO
	(n = 12)	(n = 12)	(n = 12)
**General characteristics**			
Body weight (g)	276.4±6.6	295.6±13.3	290.1±8.9
Bladder weight (mg)	111.6±3.8	105.2±5.2	114.8±3.9
Waist circumference (cm)	16.9±0.22	17.4±0.27	18.2±0.27*
Systolic pressure (mmHg)	123.6±4.1	150.1±4.6*	153.1±6.3*
Water intake	31.3±2.49	34.9±2.35	32.9±1.79
Urine output (ml/24hrs)	26.6±2.2	31.2±2.1	29.0±2.6
**Biochemistry parameters (fasting values)**			
Triglycerides (mg/dl)	34.3±2.5	86.1±4.8*	81.3±6.9*
Cholesterol (mg/dl)	45.7±2.3	66.6±3.4*	64.8±3.5*
Glucose (mM)	6.6±0.19	7.3±0.39	7.0±0.28
Insulin (mU/l)	13.9±0.69	24.2±1.94*	22.2±1.71*
†HOMA-IR	4.1±0.27	7.7±0.65*	6.9±0.53*
**Cystometry parameters**			
**Before treatment**			
Voiding pressure (mmHg)	21.5±0.9	21.8±1.2	25.0±1.1
Functional bladder capacity (ml)	1.19±0.04	1.17±0.03	0.95±0.06*
**After 5mM ATP instillation**			
Voiding pressure (mmHg)	22.3±1.1	23.1±1.2	27.7±1.4*
Voiding pressure increase (mmHg)	0.78±0.49	1.27±0.54	2.74±0.46*
Functional bladder capacity (ml)	0.89±0.08	0.79±0.04	0.52±0.04*
Lessened ICI (%)	26.0±4.6	32.9±2	46.2±2.1*
No. provoked phasic contraction(>15 cmH2O) per void	0.9±0.21	3.3±0.4*	2.1±0.27*
**Immunofluorescence stain of the bladde**r			
Nerve fiber density of suburothelium (Number per cross-section of bladder base)	537.7±36.2	489.5±40.4	578.5±31.4

Data presented as mean ± SEM and tested among groups using one-way ANOVA with Dunnett’s test.

* The Dunnett’s test showed a significant difference between the control group and other groups.

† Homeostasis model assessment of insulin resistance (HOMA-IR) = fasting plasma insulin (µ U/ml)×fasting plasma glucose (mmol/l)/22.5.

**Figure 1:**
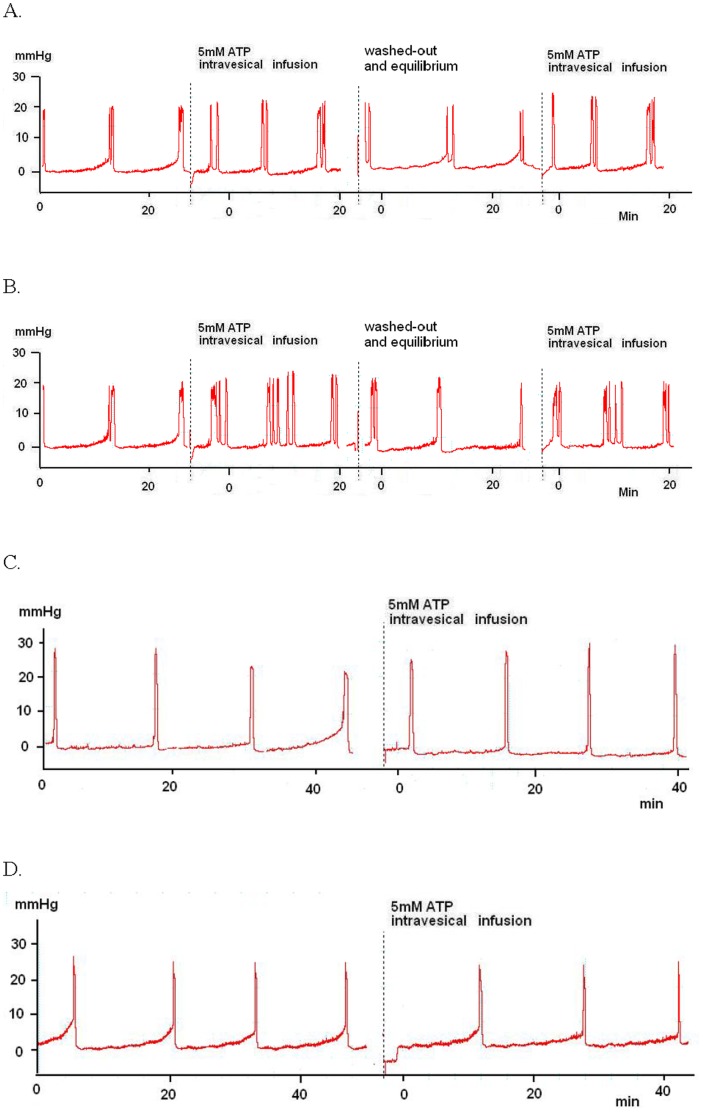
Representative cystometrographies of control and FFR responses to intravesical instillation of acidic ATP solution (5 mM, pH 3.3) alone or after pre-treatment with capsaicin. A. Reproducible effects of infused functional bladder capacity reduction were observed by intravesical infusion of acidic ATP solution in controls. B. A representative trace of infused functional bladder capacity reduction and increased provoked phasic contractions in FFRs with NDF that is repeatable during intravesical stimulation with acidic ATP solution. C. The response to acidic ATP solution stimulation of the bladder is inhibited in the control rats pretreated with capsaicin. D. Reflex micturition from acidic ATP solution stimulation of the bladder in FFRs is also inhibited by capsaicin pretreatment.

Altered C-fiber function of the skin is an indicator of idiopathic peripheral neuropathy in patients with metabolic syndrome [15]. In urodynamic studies, urothelial dysfunction and vesical C-fiber neuropathy are usually tested by a provoking test, such as an ice-water test [16]. The majority of fructose-fed rats (FFRs), an animal model of metabolic syndrome, develop DO and frequency [17], [18]. In this translational study, we stimulated the bladder mucosa of rats fed a fructose-rich diet for 3 months by instilling acidic ATP solution intravesically. Instillation of acidic ATP solution can cause afferent noise, provoke reflex micturition, and decrease cystometric bladder capacity, in which we simulate the BO condition in an animal model. The BO was determined from a lower functional bladder capacity which was induced by sensory afferent stimulation. The reduction of infused functional bladder capacity in animal experiments is so-called “bladder overactivity” in many other animal studies of the bladder [12], [19]. We hypothesized that metabolic syndrome would alter the sensory transduction mechanisms of the mucosal layer, subsequent hyperexcitability of C-fiber afferents and resulting in BO.

## Materials and Methods

### Animals

This study was performed in accordance with the guidelines of the National Science Council and Animal Protection Law by the Council of Agriculture of the Republic of China. The experimental protocol was approved by the Institutional Animal Ethics Committee of Chang Gung Memorial Hospital (Permit Number: 2008121602). All surgery was performed under urethane anesthesia, and every effort was made to minimize suffering and the number of animals used through out the experiment.

**Figure 2:**
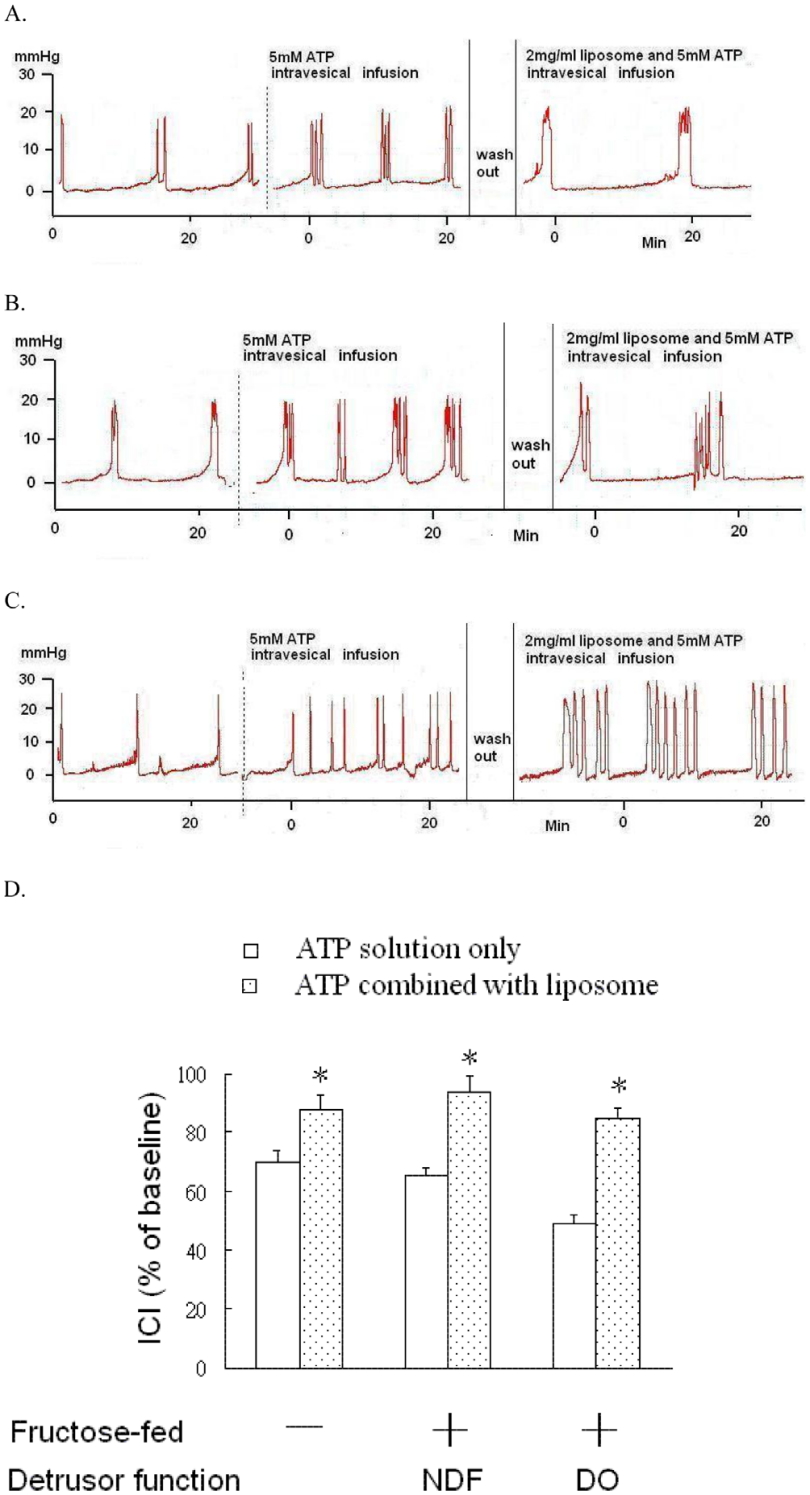
The effects of intravesical liposome administration on the response to acidic ATP solution stimulation in the bladder of controls and FFRs. Representative traces of bladder responses to acidic ATP solution instillation before and after intravesical liposome administration in the control (A), FFRs with NDF (B), and FFRs with DO (C). (D): Protective effects of liposomes on the reduction of ICI during ATP solution stimulation among groups. An asterisk indicates a significant difference between before and after intravesical liposome administration in the same group. (Paired t-test, p<0.05).

One hundred and seventy 6-week-old female Wistar rats were randomly allocated to FFR and control groups. FFRs (n = 120) were fed a fructose-rich diet (60% fructose diet, Harlan Teklad, Madison, WI), whereas control animals (n = 50) received standard rat chow for 3 months. They were maintained at a constant temperature and with a 12∶12-hour light-dark cycle. Ten FFRs were reserved for a capsaicin study. Based on the definitions provided by the International Continence Society [1], FFRs were categorized into two groups according to cystometric presentations at month 3: a group with normal detrusor function (NDF) in both the filling and voiding phase (n = 37); a group with non-voiding detrusor contraction over 4 cmH_2_O in the filling phase was recorded as DO (n = 54) [1], [20], [21]. Those that remained unclassified or unbalanced on cystometry (n = 19) were excluded from further investigation and analysis [18].

**Figure 3:**
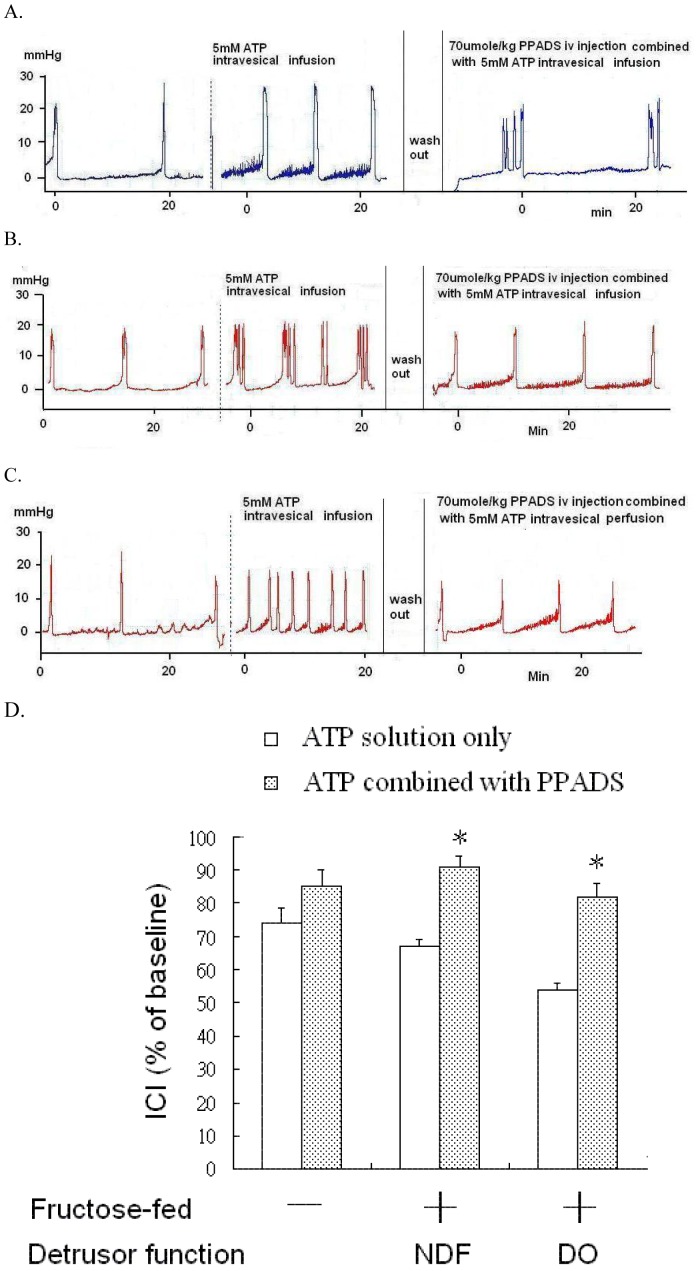
The effects of PPADS intravenous injection on the response to acidic ATP solution in the bladder of controls and FFRs. Representative traces of bladder responses to acidic ATP solution instillation before and after intravenous PPADS administration in the control (A), FFRs with NDF (B), and FFRs with DO (C). (D): Purinergic antagonist effects of PPADS on the reduction of ICI during ATP solution stimulation among groups. An asterisk indicates a significant difference between before and after intravenous PPADS administration in the same group. (Paired t- test, p<0.05).

### Micturition

After 3 months of treatment, the rats were placed in individual metabolic cages (R-2100; Lab Products, Rockville, Maryland) and the previous conditions were maintained for a familiarization period of 24 hours. After this period, a known volume of water was measured and placed in the drinking bottles of the animals, and urine was collected. The volume of liquid consumed was calculated, and urine production was measured for 3 days.

**Figure 4:**
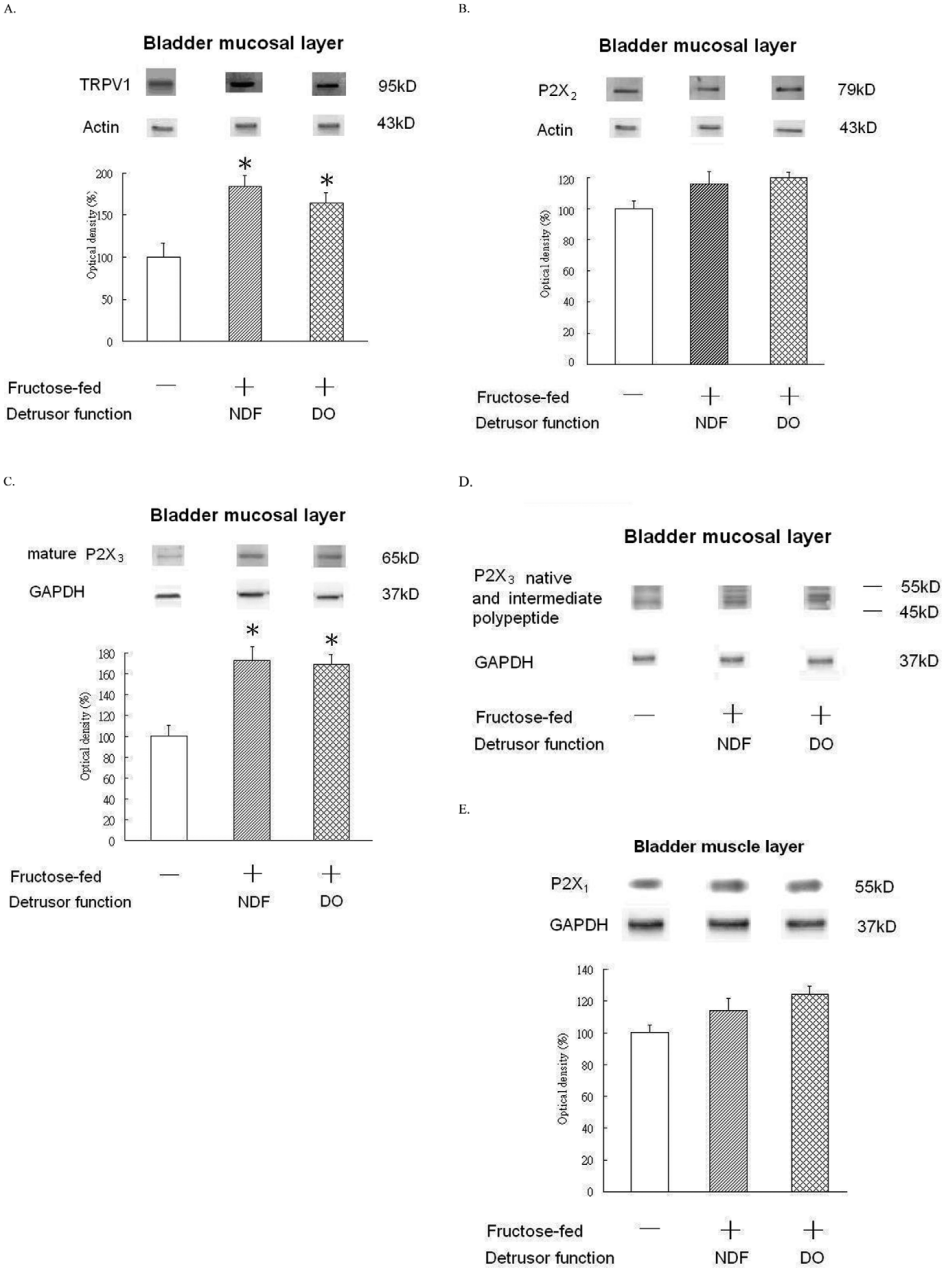
Alterations of receptor protein expression in the mucosa layer or smooth muscle layer of the bladder in controls and FFRs. Western blot analysis with specific antibodies to the TRPV1 receptor, purinergic P2X_2_ receptor, and P2X_3_ receptor of the rat mucosal layer and the purinergic P2X_1_ receptor of the rat smooth muscle layer in controls and FFRs with different in cystometric presentations. A. TRPV1 receptor: The TRPV1 antibody produced a clear single band at 95kDa. B. Purinergic P2X_2_ receptor C. Purinergic P2X_3_ mature receptor: the predominant P2X_3_ form (65kDa). D. Up-regulation of P2X3 native and intermediate polypeptides (up to 55kD) were shown in both FFR groups. E. Purinergic P2X_1_ receptor Experiments were repeated two times and representative blots are shown. Data of proteins expression (ratios of signal intensities of investigated receptors relative to β-actin or GAPDH) were calculated with 8 samples in each group. These data of Mean ± SE were standardized and expressed in percentage in which the value of the control group is treated as 100%. Theses values were shown in the bar graph. An asterisk indicates a significant difference between controls and FFR groups (One-way ANOVA with Dunnett’s test, p<0.05).

### Cystometry

All rats were anesthetized by subcutaneous injection of urethane (1.2 g/kg). Polyethelene-50 catheters were placed in the left carotid artery to measure heart rate and arterial pressure by a PowerLab® 16S system with a P23 1D transducer (Gould-Statham, Oxnard, CA), and in the left femoral vein for administration of drugs when needed. Then, the lower abdominal wall was incised and the bladder was exposed to avoid the interference of pressure changes in the abdominal cavity [20], [21]. By inserting a Polyethelene-50 catheter from the urethra to minimize the possible trauma of the bladder, the bladder catheter was connected via a T-tube to a pressure transducer and a microinjection pump (Infors AG, CH-4103, Bottmingen, Switzerland). Saline at room temperature was infused into the bladder at a rate of 0.08 ml/min and voiding pressure was recorded using a Gould polygraph (RS3400; Gould, Cleveland, OH). After starting saline infusion, we waited a minimum of 30 minutes for voiding patterns to stabilize. Thereafter, reproducible micturition cycles were recorded for a 1-hour period and used for evaluation. The rapid contraction and relaxation of detrusor was recorded as the phasic contraction, which may relate to the purinergic-induced contraction. The slow and sustained plateau contraction was recorded as the tonic contraction, which is believed to result from the cholinergic-induced contraction and takes charge of bladder emptying [22], [23]. The infused functional bladder capacity was calculated using the following equation: functional bladder capacity = inter-contraction interval (ICI) (min)×0.08 ml/min. The peak pressure of active tonic contraction in a balanced cystometry was measured for 3 times and an average value was determined as the voiding pressure. The number of provoked phasic contractions (more than 15 cmH_2_O) by acidic ATP solution stimulation was also recorded. The provoked phasic contractions observed in this study may elicit the voiding or in the voiding phase of rats.

**Figure 5:**
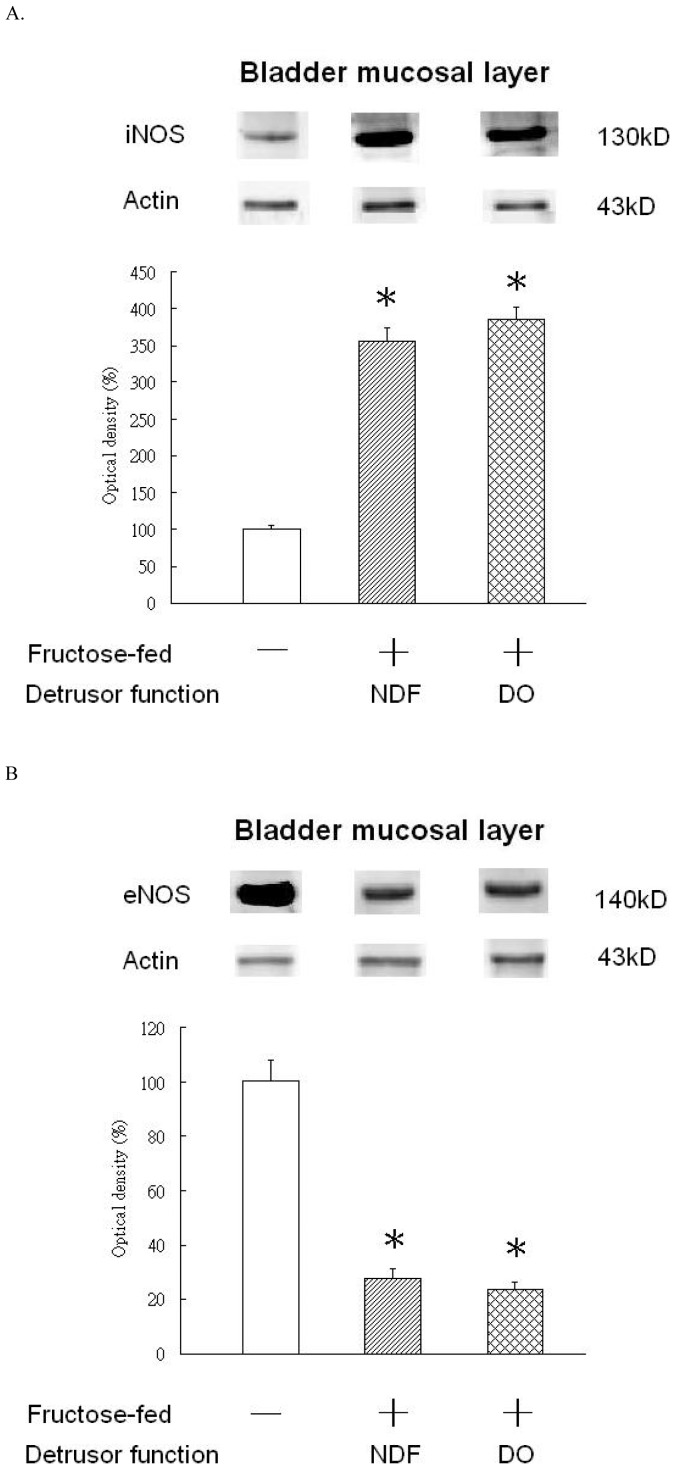
Alterations of NOS in the bladder mucosa layer of controls and FFRs. Western blot analysis with specific antibodies to iNOS and eNOS of the rat bladder mucosa layer was performed for controls and FFRs with different presentations in cystometry. A. iNOS B. eNOS Experiments were repeated two times and representative blots are shown (upper). Data of proteins expression (ratios of signal intensities of investigated proteins relative to β-actin) were calculated with 8 samples in each group. These data of Mean ± SE were standardized and expressed in percentage in which the value of the control group is treated as 100%. Theses values were shown in the bar graph (lower). An asterisk indicates a significant difference between controls and FFR groups (One-way ANOVA with Dunnett’s test, p<0.05).

### Drug Administration and Functional Experiments

After performing control cystometrographies with saline infusion, rats were subjected to one of four types of physiological experiments: (a) repeatable responses to acidic ATP solution stimulation; (b) capsaicin pretreated to inhibit the response of acidic ATP solution; (c) intravesical administration of liposome (2 mg/ml; Lipella Pharmaceuticals, Pittsburgh, Pennsylvania), a mucosal protective agent [24], [25], to partially normalize stimulation by acidic ATP solution; or (d) intravenous administration of pyridoxal 5-phosphate 6-azophenyl-2′,4′-disulfonic acid (PPADS, 70 µmol/kg; Sigma), a commonly used noncompetitively antagonist for heteromeric P2X_2_/P2X_3_, homomeric P2X_3_ and P2X_1_ receptors [26], to reverse the stimulation by acidic ATP solution. ATP (Sigma) was dissolved in distilled water (5 mM, pH 3.3).

The repeatability of acid ATP solution responses was determined by infusing ATP solution intravesically in rats (6 controls and 6 NDF FFRs) to evoke BO (reduced maximum functional bladder capacity) and provoke phasic contractions. Rats were infused with ATP solution for 1.5hours, starting after the baseline cystometric data was collected using normal saline. To ensure stable voiding patterns, data from the initial 20 min of ATP solution instillation was not analyzed [11]. Following the stabilization period, data from 1-h of reproducible micturition cycles was collected and used for evaluation. After washing with normal saline and a 1-h equilibrium period, we repeated the ATP solution instillation procedure. The ATP solution was administered to examine whether ATP solution at low concentration (5 mM) and at pH 3.3 could induce repeatable BO and phasic contractions in the study animals.

In a different group of rats (6 controls and 6 FFRs), ATP solution was instilled intravesically after pretreatment with systemic capsaicin. Capsaicin (Sigma) was administered to rats in a solution (20 mg/ml) given subcutaneously in divided doses on 2 consecutive days: 25 and 50 mg/kg on the first day and 50 mg/kg on the second day [11]. Cystometry and ATP solution stimulation as in the previous experiment were performed four days after the first injection. We performed an eye wipe test on each rat to evaluate the effectiveness of capsaicin pretreatment [11].

For the third and fourth studies, ATP solution stimulation was performed in rats (6 controls, 6 FFRs with NDF, and 6 FFRs with DO, each study) using the same procedure as in the initial ATP solution stimulation tests, except that liposomes and PPADS were used to interfere with the effects of acidic ATP solution. In the third study, liposomes were added to the ATP solution that was instilled intravesically in the second course of stimulation. In the fourth study, the P2X receptor antagonist, PPADS, was injected intravenously 5 minutes before the second course of ATP solution infusion [11].

### Determination of Functional Protein Levels and Suburothelial Nerve Fiber Density

We used 12 rats per group. After performing control cystometrographies with saline infusion and one course of ATP solution stimulation (1.5 hours), rats were sacrificed using an overdose of urethane. Blood samples were collected for biochemical analysis. Insulin resistance was calculated by homeostasis model assessment of insulin resistance using the equation, fasting plasma insulin in µ U/ml×fasting plasma glucose in mmol/l/22.5. Bladder bodies were assayed using western blot and bladder bases were prepared for immunofluorescence staining.

The mucosa and detrusor muscle layer of the bladder body were obtained using a microdissection technique [18], [27]. To avoid cross-contamination of the 2 layers, the proficiency of separation was assessed histologically. The expression levels of TRPV1 receptor, P2X_2_ receptor, P2X_3_ receptor, inducible nitric oxide synthase (iNOS) and endothelial nitric oxide synthase (eNOS) in the bladder mucosa, and P2X_1_ receptor in the muscle layer were evaluated by western immunoblotting, as previously described [19]. Briefly, alternative samples were homogenized in protein extraction solution before sonication (Sonics vibra-Cell™) and purification. Total protein was measured using the Pierce 660 nm Protein assay (Thermo). Sodium dodecyl sulfate-polyacrylamide gel electrophoresis was performed using the Laemmli buffer system. Antibodies raised against TRPV1 receptor (Alomone, Israel; 1∶1000 dilution), P2X_2_ receptor (Alomone, Israel; 1∶500 dilution), P2X_3_ receptor (Neuromics, MN; 1∶500 dilution), P2X_1_ receptor (Alomone, Israel; 1∶500 dilution), iNOS (Cayman Chemical, Michigan; 1∶500 dilution), eNOS (BD Biosciences, CA; 1∶1000 dilution), GAPDH (Millipore, CA; 1∶10000 dilution ) and β-actin (Millipore, CA; 1∶10000 dilution) were used. Quantitative analysis was performed using LabWorks™ image acquisition and analysis software. According to the product information of manufacturer, the molecular weights of proteins were checked respectively.

Alternative bladder base samples were frozen and mounted in Tissue-Tek® O.C.T.™ mounting medium. Twenty serial tissue sections (10 µm) were cut on a cryostat, beginning at the level of the vesicoureteral junction and mounted on Superfrost® slides. Immunostaining with an antibody against pan-neuronal marker protein gene product 9.5 (Millpore, CA) was performed on the 12^th^ and 13^th^ sections of each specimen. Labeled nerve fibers and photomicrographs were captured using a fluorescence microscope and 5 fields at×200 magnification were visualized using Image-Pro® 6.1. Recombination of photomicrographs was performed to show the number of positive immunostaining nerve spots in the suburothelial layer of the whole transverse section. The suburothelial layer contained abundant C-fiber afferents to modulate sensation [28], [29].

### Statistical Analysis

All data are presented as the mean ± standard error. Data were subjected to paired t-tests or 1-way ANOVA with Dunnett’s post hoc test for multiple comparisons. For all statistical tests, p<0.05 was considered significant.

## Results

### General Characteristics and Cystometric Studies

Table 1 lists the general characteristics of all experimental groups and their responses to acidic 5 mM ATP solution instillation. After 3 months of fructose feeding, FFR groups manifested features of metabolic syndrome, including a significant increase in the fasting insulin, triglyceride, and cholesterol levels of the blood. Systolic pressure and insulin resistance also increased in the FFR groups. A significant increase in waist circumference was observed in DO FFRs. There was no significant difference in the nerve density of the suburothelial layer between controls and either FFR group.

NDF FFRs showed similar voiding behavior to controls, except for an increase in provoked phasic contractions after ATP solution instillation of the bladder (Table 1). DO FFRs had lower bladder capacity than controls when infused with normal saline. During instillation with ATP solution, the DO FFRs had significantly greater voiding pressure, lessened bladder capacity, and more phasic contractions than controls.

### Responses to Acidic ATP Solution and Effects of Capsaicin-pretreatment

When ATP solution was infused intravesically, bladder capacity decreased and phasic contractions increased compared to infusion with normal saline in both controls and FFRs. After washing with saline for 1 h, re-infusion with ATP solution produced responses that were similar to those seen during the first infusion of ATP solution (Fig. 1, A and B). However, capsaicin-treated rats did not differ in their responses to saline solution and ATP solution (Fig. 1, C and D). This indicates that C- fiber desensitization by capsaicin pretreatment suppressed acidic ATP solution-initiated BO and reflex micturition [11].

### Effects of Liposome and P2X Receptor Antagonist

The addition of 2 mg/mL liposome to the ATP solution partially reversed the ATP solution-induced response in rats (Fig. 2). PPADS (70 µmole/kg, IV) decreased the ATP solution-induced response in FFRs (Fig. 3).

### Assessment of TRPV1, P2X_2_, P2X_3_, iNOS and eNOS in the Bladder Mucosal Layer and P2X_1_ in the Bladder Detrusor Muscle Layer

Representative western blotting results and statistical comparisons for the expression of functional proteins in the mucosa and detrusor of the control and FFR groups are shown in Figures 4 and 5. Both FFR groups showed significant higher TRPV1, total P2X_3_ and iNOS protein expression, but lower eNOS protein expression, as compared to controls. P2X_2_ and P2X_1_ protein expression was not significantly different between controls and FFR groups.

## Discussion

The results of this study show that rats fed a high fructose diet can alter the sensory function of bladder mucosa. This dysfunction includes over-expression of TRPV1 and total P2X_3_ receptors during the stimulation of acidic ATP solution, which may enhance iNOS expression, activate the C-fiber afferents, provoke phasic contractions, and induce BO. As a result of down-regulation, the decreased level of eNOS protein in the bladder mucosa of FFRs may be unable to suppress the BO and phasic contractions. However, despite similar dysregulation of these functional proteins in the bladder mucosa in the two FFR groups, the responses to the acidic ATP solution differed between the NDF group and the DO group of FFRs. Compared to controls, DO FFRs had a significant reduction of functional bladder capacity and increased bladder voiding pressure during ATP solution stimulation, reflecting the instability of the detrusor in these rats. Therefore, when the bladder mucosa is in contact with noxious substances or elevated ATP concentrations, the character of DO may exacerbate the BO condition in FFRs.

Increasing evidence links features of metabolic syndrome with OAB [3]. Several animal models of metabolic syndrome indicate that DO is a popular urodynamic characteristic of metabolic bladder dysfunction [21], [30], [31]. The pathogenesis of abnormal vesical sensory-motor function in metabolic syndrome is not completely understood and most likely has a multi-factorial origin. Central obesity is a key feature of metabolic syndrome and may induce OAB in humans [32]. Some researchers have suggested that excess body weight increases the abdominal pressure and this pressure increase exacerbates detrusor instability, and resulting in urgent incontinence [33]. However, in this study, DO FFRs had larger waist circumferences than controls, but did not have greater body weights. Generally speaking, the accumulation of visceral fat due to metabolic syndrome will lead to over-expression of adipokines, such as resistin, IL-6, and TNF-α, resulting in diminish eNOS activity, less NO generation and endothelial dysfunction [34]. Treating the metabolic features by using INT-747, a farnesoid X receptor agonist, improves the bladder RhoA/Rho kinase (ROCK) overactivity of high fat diet fed rabbits [35]. We propose that that the dysregulated metabolism of adipose tissue plays a role in the development of DO, and that DO does not result simply from the physical effects of obesity.

The data from our functional experiments suggest that activation of “neuron-like” properties of bladder mucosa and subsequent C- fiber hyperactivity contribute to the initiation of BO and the emergence of phasic contractions. The sensitization of silent C-fibers has been implicated in the etiology of OAB in humans [2]. In animals pretreated with capsaicin to desensitize the C-fibers we found that the effect of intravesical acidic ATP solution was suppressed in normal rats as well as FFRs. These results provide indirect evidence that sensitization of C afferents plays a major role in the BO induced by intravesical administration of ATP. Changes of the bladder microenvironment (acidic, toxic, or inflammation) might activate the C afferent fibers and cause BO. The initiation of acidic ATP solution stimulation might be through the urothelium, as the reduction in functional bladder capacity was not as marked when acidic ATP solution was co-administered with liposome as when acidic ATP solution was administered alone. Intravesical liposome exposure can create a molecular film on the urothelium and improve the barrier function of bladders that have been pre-treated with acidic ATP solution [24], [25]. The experiments of capsaicin pre-treatment test and liposome protective test demonstrated the action of acidic ATP solution might be due to impairment of the urothelial barrier function and activation of suburothelial C-afferent fibers.

Over-expression of the TRPV1 and P2X_3_ receptors in the mucosal layer plays a crucial role in the initiation of provoked phasic contractions and BO in FFRs groups. Suburothelial C-fibers can be divided to capsaicin-sensitive and capsaicin-insensitive subtypes [36]. Yu et al. reported that activation of capsaicin-sensitive C- fibers, which is believed to occur via TRPV1 receptors associated with ATP release and inflammatory status, can facilitate reflex phasic contractions in rats [37]. Researchers have also reported that activation of capsaicin-insensitive C-fibers via the P2X_3_ receptor alone will reduce cystometric bladder capacity [36]. It seems that the increase in provoked phasic contractions of FFRs were not due to the direct effects of the purinergic agonist on bladder smooth muscle. This is because the expression of P2X_1_ receptors in the smooth muscle of FFRs was not different from controls. The estimated suburothelial nerve density also did not differ between controls and FFRs. It is therefore more likely that the increase in provoked phasic contractions observed in the FFRs resulted from an increase in the excitability of capsaicin-sensitive C-fibers via the over-expression of TRPV1 receptors. Therefore, we suggested that the significant increase of provoked phasic contractions during the acidic ATP solution stimulation might be due to the increased expression of TRPV1 receptors in the bladder mucosa of FFRs. In addition, this finding of unchanged suburothelial nerve fiber density of FFRs suggested that the bladder mucosa sensory dysfunction occur in the early stage of peripheral neuropathy of metabolic syndrome. Clinically, in the development of pre-diabetes peripheral neuropathy the functional defects may precede structural changes [15]. Therefore, this is a crucial timing for assessing interventions to prevent or delay progress of neuropathy for victims of metabolic syndrome at the early stage.

Our findings in the current study suggest that sensory dysfunction of bladder mucosa may trigger BO in DO FFRs and might contribute to the exacerbation of OAB further. The functional bladder capacity of NDF FFRs was not significantly reduced, even though it had indications similar to the dysregulation of sensory receptors and enzymes in the bladder mucosa and, when stimulated by ATP solution, demonstrated similar phasic contractions to DO FFRs. It is possible that the instability of the detrusor in DO FFRs might have a lower action potential threshold facilitating reflex micturition and reducing bladder capacity. Usually, the OAB patients having DO are likely to experience more severe urgency than those have not [38]. Urothelium involves in sensory mechanisms of the bladder and can release chemical mediators, including ATP, NO, prostaglandin E2, and nerve growth factor [2]. Bladder mucosa layer along with C afferents plays an important role in the sensory transduction mechanisms modulating micturition, particularly in conditions of increased sensory nerve transmission following acute chemical irritation, chronic inflammation, or metabolic derangement, in which conditions would modulate the micturition reflex and cause bladder overactivity. Munoz et al reported that intravesical ATP stimulation on bladder mucosa may increase sensory activity at the spinal cord level and regulate the bladder overactivity [12]. In vitro studies, scientists also reported the spontaneous phasic activity of the detrusor muscle was associated with myogenic hyperexcitability of muscle strips in unstable bladders [39]. It has been shown that P2X receptor-mediated smooth muscle cell contraction involves Ca2+ sensitization via activation of the RhoA/ROCK pathway [40], [41]. Smooth muscle contraction is highly dependent on ROCK-catalyzed phosphorylation of myosin phosphatase-targeting subunit at T855, leading to inhibition of myosin light chain phosphatase and increased myosin regulatory light chain phosphorylation [42]. Morelli et al reported that rabbits fed with high fat diet may develop RhoA/ROCK overactivity in the detrusor, which disclosed an important molecular mechanism underlying metabolic bladder dysfunction [35]. Further study with the analysis of protein extracts from rat bladders for ROCK activity by evaluating the amount of ROCK-catalyzed phosphorylation of myosin phosphatase-targeting subunit at T855 would be interesting. In addition, without a provoking test, NDF FFRs show normal voiding behavior despite of obvious sensory dysfunction of bladder mucosa. This result may explain why some OAB patients presented with NDF in urodynamic studies [38]. However, if the microenvironment of the bladder mucosa in these patients were to change, suburothelial C- fiber hyperactivity would occur.

NO released from urothelium is involved in the activity of underlying bladder afferent nerves or the spontaneous activity of smooth muscle [2], [4]. Our results indicate that NO derived from eNOS and iNOS proteins in the mucosal layer have opposing functions in BO. Two major isoforms of NOS with pathophysiological functions in the bladder have been reported: eNOS and iNOS [43]. eNOS is expressed constitutively and its activity is controlled by calmodulin binding in a Ca^2+^ concentration-dependent manner. iNOS is inducible and not regulated by calmodulin. It is known that protons can activate TRPV1 receptors to release ATP and inducible NO [44]. These substances can irritate C-fiber afferents, causing BO and reflex micturition [2], [4]. Moreover, inflammation can trigger the expression of iNOS in the bladder and subsequently augment the contractile response by activating the protein kinase C pathway [45]. Systemic inflammation is a part of the pathophysiology of metabolic syndrome and precipitates the development of DO in FFRs, as we reported previously [17]. On the other hand, animal studies have demonstrated that deficient constitutive expression of NO can induce DO and bladder remodeling [43], whereas NO substrate supplements, such as L-arginine, can reduce Aδ and C-afferent activity in rats [46].

There are some limitations in this study. This study did not measure the rat’s sensation directly. Empirically, the measurement of reduced functional bladder capacity, so-called “bladder overactivity”, is deduced to be secondary to the sensory stimulation of rats’ bladder mucosa. Although BO is usually present in DO bladders, BO may refer to a group of patients with reduced functional bladder capacity, but with or without DO appeared in urodynamic tracing. This response in cross-talk between sensory and contractile pathways may depend on the varied bladder dysfunction in different diseases. The urinary symptom of urgency reported from humans with OAB may not be applied to an animal model. Another limitation is the lack of presenting naïve expression of functional proteins in the current study. Further study with the measurement of protein expressions without any bladder manipulation is important and might reflect the real changes of FFR bladders.

### Conclusion

After 3 months, FFRs generally had dysregulation of sensory receptors and enzymes in the bladder mucosa, including over-expression of TRPV1, total P2X_3_, and iNOS proteins under acidic ATP stimulation. These changes may contribute to the emergence of phasic bladder contractions during stimulation by acidic ATP solution. Down-regulation of eNOS protein in the bladder mucosa of FFRs may result in a deficiency of constitutive NO, thereby limiting suppression of C-afferent hyperactivity. Dysregulation of sensory transduction mechanisms of the bladder mucosa and DO causing by metabolic syndrome are easier to elicit BO when the mucosa is subjected to certain stimuli. Our findings may provide insight into the mechanism involved in the pathophysiology of OAB in patients with metabolic syndrome.
